# Exploring the association between circulating endothelial protein C receptor and disease activity of rheumatoid arthritis in a pilot study

**DOI:** 10.1093/rap/rkae096

**Published:** 2024-08-06

**Authors:** Meilang Xue, Haiyan Lin, Tom Lynch, Lara Bereza-Malcolm, Premarani Sinnathurai, Ranjeny Thomas, Helen Keen, Catherine Hill, Susan Lester, Mihir Wechalekar, Lyn March

**Affiliations:** Sutton Arthritis Research Laboratory, Sydney Musculoskeletal Health, Kolling Institute, Faculty of Medicine and Health, The University of Sydney, Sydney, NSW, Australia; The Australian Arthritis and Autoimmune Biobank Collaborative (A3BC), Sydney Musculoskeletal Health, Kolling Institute, Faculty of Medicine and Health, The University of Sydney and the Northern Sydney Local Health District, Sydney, NSW, Australia; Sutton Arthritis Research Laboratory, Sydney Musculoskeletal Health, Kolling Institute, Faculty of Medicine and Health, The University of Sydney, Sydney, NSW, Australia; The Australian Arthritis and Autoimmune Biobank Collaborative (A3BC), Sydney Musculoskeletal Health, Kolling Institute, Faculty of Medicine and Health, The University of Sydney and the Northern Sydney Local Health District, Sydney, NSW, Australia; The Australian Arthritis and Autoimmune Biobank Collaborative (A3BC), Sydney Musculoskeletal Health, Kolling Institute, Faculty of Medicine and Health, The University of Sydney and the Northern Sydney Local Health District, Sydney, NSW, Australia; Sutton Arthritis Research Laboratory, Sydney Musculoskeletal Health, Kolling Institute, Faculty of Medicine and Health, The University of Sydney, Sydney, NSW, Australia; The Australian Arthritis and Autoimmune Biobank Collaborative (A3BC), Sydney Musculoskeletal Health, Kolling Institute, Faculty of Medicine and Health, The University of Sydney and the Northern Sydney Local Health District, Sydney, NSW, Australia; The Australian Arthritis and Autoimmune Biobank Collaborative (A3BC), Sydney Musculoskeletal Health, Kolling Institute, Faculty of Medicine and Health, The University of Sydney and the Northern Sydney Local Health District, Sydney, NSW, Australia; Department of Rheumatology, Royal North Shore Hospital, Syndey, NSW, Australia; Frazer Institute, Translational Research Institute, The University of Queensland, Brisbane, QLD, Australia; Medical School, The University of Western Australia, Perth, WA, Australia; Department of Rheumatology, Fiona Stanley Hospital, Murdoch, WA, Australia; Adelaide Medical School, The University of Adelaide, Adelaide, SA, Australia; Rheumatology Research Group, Paediatrics, and Paediatric Rheumatology, Basil Hetzel Institute and The Queen Elizabeth Hospital, Adelaide, SA, Australia; Adelaide Medical School, The University of Adelaide, Adelaide, SA, Australia; Rheumatology Research Group, Paediatrics, and Paediatric Rheumatology, Basil Hetzel Institute and The Queen Elizabeth Hospital, Adelaide, SA, Australia; Rheumatology Synovial Tissue Translational Research Group, Flinders University, Adelaide, SA, Australia; Rheumatology Unit, Flinders Medical Centre, Adelaide, SA, Australia; The Australian Arthritis and Autoimmune Biobank Collaborative (A3BC), Sydney Musculoskeletal Health, Kolling Institute, Faculty of Medicine and Health, The University of Sydney and the Northern Sydney Local Health District, Sydney, NSW, Australia; Department of Rheumatology, Royal North Shore Hospital, Syndey, NSW, Australia

**Keywords:** endothelial protein C receptor, RA, single nucleotide polymorphism, T cells

## Abstract

**Objectives:**

To investigate whether circulating endothelial protein C receptor (EPCR) is associated with disease activity and inflammatory markers in rheumatoid arthritis.

**Methods:**

Thirty-eight RA patients and 21 healthy controls (HC) were recruited via the A3BC biobank. Peripheral blood mononuclear cells and plasma were isolated from the blood of these participants. Plasma soluble (s)EPCR, IL-6, IL-17 and sCD14 were measured by enzyme-linked immunosorbent assay, cell membrane-associated (m)EPCR by flow cytometry; *EPCR* gene H3 single nucleotide polymorphism (SNP), which contributes to high plasma sEPCR levels, by PCR and DNA sequencing. Data were analysed using FlowJo10 and GraphPad Prism 10.

**Results:**

RA patients had higher levels of mEPCR on T cells and plasma sEPCR compared with HC. No difference in the *EPCR* gene H3 SNP G genotype frequency was found between RA and HC. This SNP was significantly correlated with higher sEPCR levels in HC but not in RA patients. In RA, plasma sEPCR levels were positively correlated with IL-6, IL-17, sCD14, anti-CCP and rheumatoid factor. In contrast, mEPCR levels on T cells and natural killer cells (NK) were inversely associated with disease activity measures including 28/66 swollen joint count, 28/68 tender joint count and/or DAS28-CRP/ESR scores, and positively correlated with *EPCR* gene H3 SNP, which was also correlated with lower disease activity measures in RA.

**Conclusion:**

Our findings suggest that EPCR may play an important role in RA, with plasma sEPCR being potentially associated with inflammatory markers and mEPCR and the *EPCR* gene H3 SNP possibly related to disease activity measures.

Key messages

- RA patients had higher levels of T cell mEPCR and plasma sEPCR.
- Circulating sEPCR levels were associated with inflammatory markers in RA.
- mEPCR and *EPCR* gene H3 SNP were inversely related to disease activity measures in RA.

## Introduction

The endothelial protein C receptor (EPCR) was first discovered on the surface of endothelial cells as a receptor specific to anticoagulant protein C (PC) and its activated form APC [1]. When EPCR binds with PC, it promotes the generation of APC. The free APC inactivates factors (F)Va and FVIIIa to reduce the thrombin production, regulating the anti-coagulation. Whereas APC that binds to EPCR exerts anti-inflammatory and cytoprotective effects [2]. Over time, EPCR has also been discovered in various other cell types, including myeloid cells [3], joint synovial fibroblasts [4] and even human platelets [5]. Additionally, EPCR may serve as a potential marker for various types of stem cells, including haematopoietic, epithelial, neuronal, multipotent progenitors, breast cancer, and skin epidermal stem cells [6–13]. Subsequently, EPCR has been found to interact with many other ligands, such as FVII, plasmodium falciparum erythrocyte membrane protein 1, secretory group V phospholipases A2 (sPLA2V), γδ T cells, and autoantibodies to phospholipids (aPLs) [14–16], eliciting ligand-specific functions [3]. Furthermore, EPCR resembles major histocompatibility complex class I/CD1 family proteins [1], which are crucial antigen-presenting molecules for immune surveillance by T cells, displaying an immune regulatory function [3, 17]. For example, EPCR on murine T cells can suppress Th17 pathogenicity [17]; and the binding of EPCR to γδ T cells may play an important role in monitoring the endothelium for viral infections or malignancies [18, 19].

In both physiological and pathological conditions, the cell membrane-associated (m)EPCR can detach from the cell surface and exists as a soluble form known as sEPCR in the bloodstream. Elevated levels of sEPCR in plasma are associated with a particular variation in the *EPCR* gene, called single nucleotide polymorphism (SNP) H3 G genotype [20]. In addition, inflammatory mediators including TNF-α, IL-1β, thrombin and lipopolysaccharide can induce the shedding of EPCR [21, 22]. Although sEPCR can bind to its ligands, it cannot trigger downstream signalling pathways like its membrane-bound counterpart, mEPCR [3].

Higher levels of EPCR have been associated with a wide range of autoimmune and infectious diseases, including cancers [3, 23, 24], severe lung inflammation, diabetes and lupus [3], severe malaria [25] and foetal growth restriction [26]. Research on mice has shown that EPCR deficiency can reduce inflammation caused by bacterial-induced lung injury [27] and joint bleeding-induced pathology [28]. Moreover, in patients with psoriasis, high levels of mEPCR on circulating T cells are positively correlated with disease severity [29]. In mice, blocking EPCR has been found to prevent lupus and antiphospholipid syndrome [14]. These findings suggest that EPCR may play a crucial role in the development and progression of these diseases.

RA is an autoimmune disease associated with joint inflammation and destruction [30]. In RA, the excessive activation of immune cells contributes to disease pathogenesis by the production of autoantibodies, such as RF and anti-CCP antibodies (anti-CCP) and inflammatory cytokines [31, 32]. Our previous research has shown that EPCR is overexpressed in RA joint synovium [4, 33], and severe deficiency of this receptor prevents inflammatory arthritis in mice [34]. In RA patients, however, circulating EPCR including sEPCR and mEPCR, EPCR H3 SNP G genotype and their associations with disease activity and inflammatory markers/cytokines are unknown.

## Methods

### Participants

Thirty-eight established RA patients and 21 healthy controls (HC) were included in this study. RA patients fulfilled the 2010 ACR/EULAR criteria [35]. HC had no known history of autoimmune diseases. Peripheral blood mononuclear cells (PBMC), plasma, patient demographic and clinical parameters including age, gender, disease duration, the CRP, ESR, anti-CCP (positive/negative), RF (positive/negative) and disease activity measures such as 28/66 Swollen Joint Count (SJC); 28/68 Tender Joint Count (TJC); DAS28-CRP, DAS28-ESR were collected from The Australian Arthritis and Autoimmune Biobank Collaborative (A3BC) at a single timepoint only. At the time of blood sampling, 19 patients were known to be on oral conventional synthetic DMARDs (csDMARDs) such as methotrexate (Methoblastin, Ledertrexate), 22 patients were on biologic or targeted synthetic (b/ts)DMARDs, 11 patients were taking glucocorticoids (10 on prednisone, 1 on hydrocortisone)—Supplementary Tables S1 and S2, available at *Rheumatology Advances in Practice* online, list the relevant characteristics of RA patients.

The use of human specimens and patients’ demographic and clinical parameters was approved by the Northern Sydney Local Health District Human Research Ethics Committee (RESP/18/058) and A3BC (AccessID: ResID1-PID2). All patients provided their written informed consent.

### ELISA

Plasma IL-6, IL-17, sCD14 and sEPCR were measured using ELISA kits (R&D Systems, Minneapolis, USA) according to the manufacturer’s instructions.

### *EPCR* gene SNP detection

Genomic DNA was isolated from the whole blood. DNA sequence of EPCR was obtained from GenBank. The primer sequences used to target the EPCR gene H3 4600A/G (rs867186, 290 bp) were: Forward 5′-CCTACACTTCGCTGGTCCTGGGCGTCCTGGTCTGC-3′; reverse 5′-CAAGTACTTTGTCCACCTCTCC-3′. PCR was performed using a thermal cycler (T100 Thermal Cycler, Bio-Rad Laboratories Pty Ltd, South Granville NSW, Australia) and the resultant PCR products purified and sequenced by Sanger sequencing at The Australian Genome Research Facility. All samples were duplicated to confirm the sample quality and sequencing accuracy. The consistency rates between the duplicated SNPs across all samples were greater than 99%. In addition, to minimize errors, all genotyping data was double scored by two independent researchers. The SNP was analysed using SnapGene viewer (Dotmatics, Boston, MA, USA).

### Flow cytometry

Antibody panel including CD3-BV510, CD4-BB700, CD8-APC-H7, CD11c-PECF594, CD14-BV786, CD16-PE-Cy™7, CD19-APC-R700, CD56-BV711, HLADR-BV605, EPCR-PE (BD Biosciences, North Ryde, NSW, Australia) was used to identify CD3^+^, CD4^+^ and CD8^+^ T cells, total CD19^+^ B cells, natural killer cells (NK), dendritic cells (DC), monocyte subsets and EPCR expression on these cells by a flow cytometry (LSRFortessa™ flow cytometer, BD Biosciences). Live/dead discrimination was performed by 7-AAD staining. Data were analysed using FlowJo V10.9 software (BD Biosciences).

### Quality control steps

Sample quality (FlowAI) and the normality of flow cytometric data were examined using FlowJo V.10.9 (BD Biosciences) and Prism GraphPad 10 (GraphPad Software, Boston, MA, USA). Only good events were analysed. Furthermore, only samples with at least 50000 single live cells were included in subsequent analyses to ensure that at least 100 cell events were identified in every cell subset. Finally, to minimize potential batch effects, one standardized sample with the same conditions was included in each batch.

### Clustering with FlowSOM and Cluster Explorer and visualization with t-SNE

The automated clustering steps were performed with *FlowSOM* and *Cluster Explorer* using FlowJo Plugins *in FlowJo 10*. The similarity of single cells in two-dimensional space was visualized with t-SNE. Marker enrichment modelling (MEM), an analysis method for automatically generating quantitative labels for cell populations, was used for quantitative comparison of clusters.

### Statistical analysis

Statistical analysis was performed using GraphPad Prism 10. To compare different groups, the Wilcoxon matched-pairs signed rank test or Mann–Whitney *U* test was employed, while non-parametric Spearman’s rank correlation coefficient was used to determine any correlations between variables. A two-sided *P *<* *0.05 denoted statistical significance.

## Results

A total of 21 HC (49 ± 13.3 years old, 13 females) and 38 RA patients were recruited for this study. The demographic and clinical parameters of RA patients are listed in Supplementary Tables S1 and S2, available at *Rheumatology Advances in Practice* online. Within RA, male patients had a significantly shorter disease duration when compared with females (1.2 ± 1.8 vs 14.8 ± 11.4 years old, *P* < 0.0001). Numbers included in the exploratory analyses depended on the completeness and availability of blood samples and clinical data.

### Circulating levels of EPCR in patients with RA were higher when compared with HC

**Figure 1.**
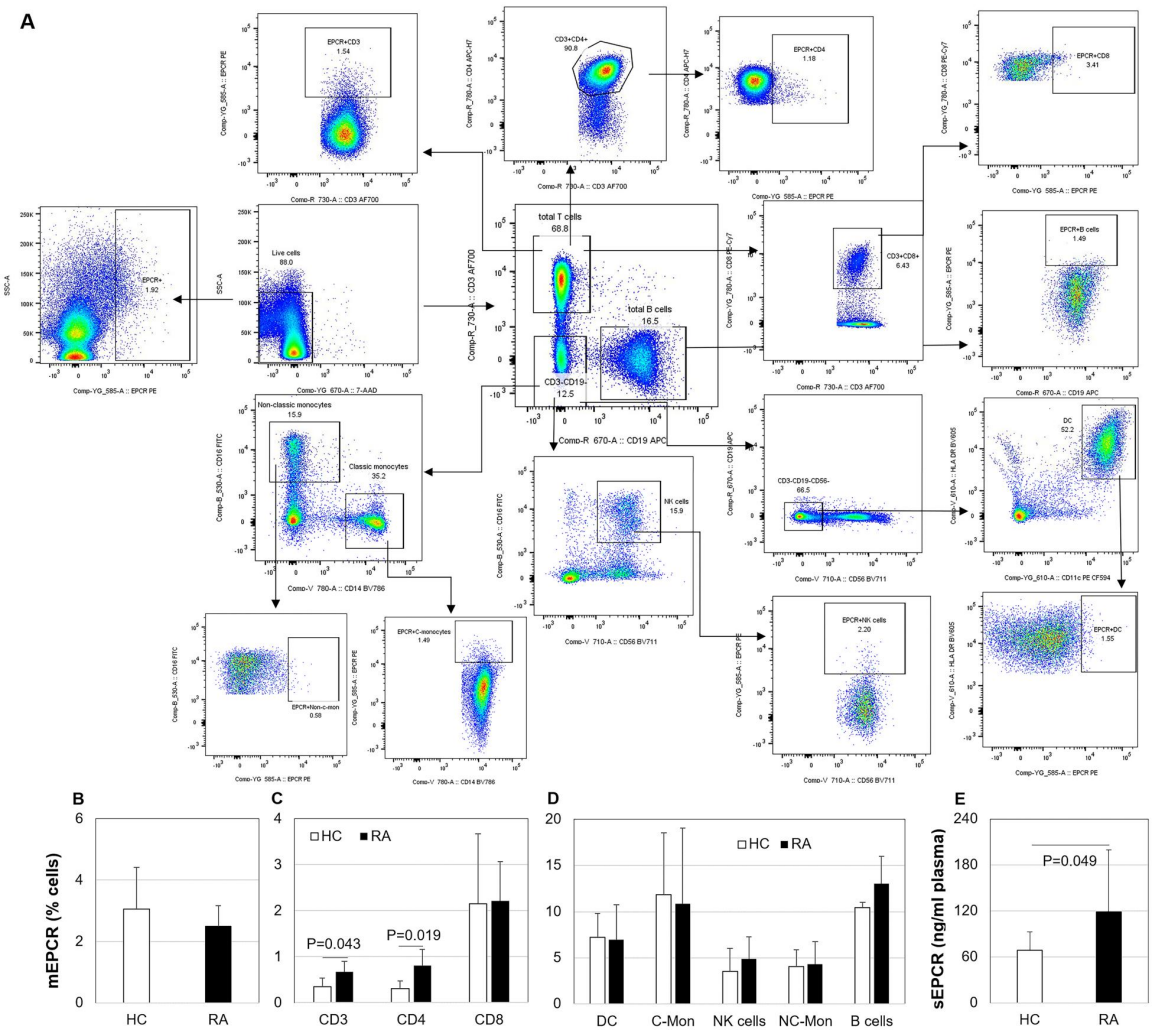
Immune cell membrane-bound (m)EPCR in healthy controls (HC) and patients with RA. Immune cell mEPCR within PBMC from 10 age and gender-matched HC and RA patients were detected by flow cytometry. (A) The gating strategies for flow cytometric detection. (B–D) The mEPCR levels on PBMC (B); T cells (C) and other immune cells (D) including dendritic cells (DC), classical monocytes (C-Mon), natural killer (NK) cells, non-classical monocytes (NC-Mon) and B cells. (E) Plasma sEPCR levels in RA and HC, detected by enzyme-linked immunosorbent assay. Significance was detected by Wilcoxon matched pairs signed rank test. Data on the graph are shown as mean (s.d.) (*n* = 10)

PBMC from 34 RA patients and 10 HC were analysed by flow cytometry. Figure 1A illustrated the gating strategies used to detect CD3^+^, CD3^+^CD4^+^ and CD3^+^CD8^+^ T cells, B cells, NK cells, DCs and monocyte subsets and their cell surface EPCR levels. There was no difference in mEPCR levels on PBMC between age and gender-matched HC and RA (*n* = 10, 46.25 ± 13.1 years old and contained 1 male) (Fig. 1B). Within the PBMC, mEPCR levels on RA CD3^+^ and CD3^+^CD4^+^ T cells were significantly higher than HC cells (Fig. 1C), but no differences on DC, classical and non-classical monocytes, NK cells and B cells were observed (Fig. 1D). This study also confirmed that RA patients had higher levels of sEPCR in plasma compared with HC (Fig. 1E).

**Figure 2.**
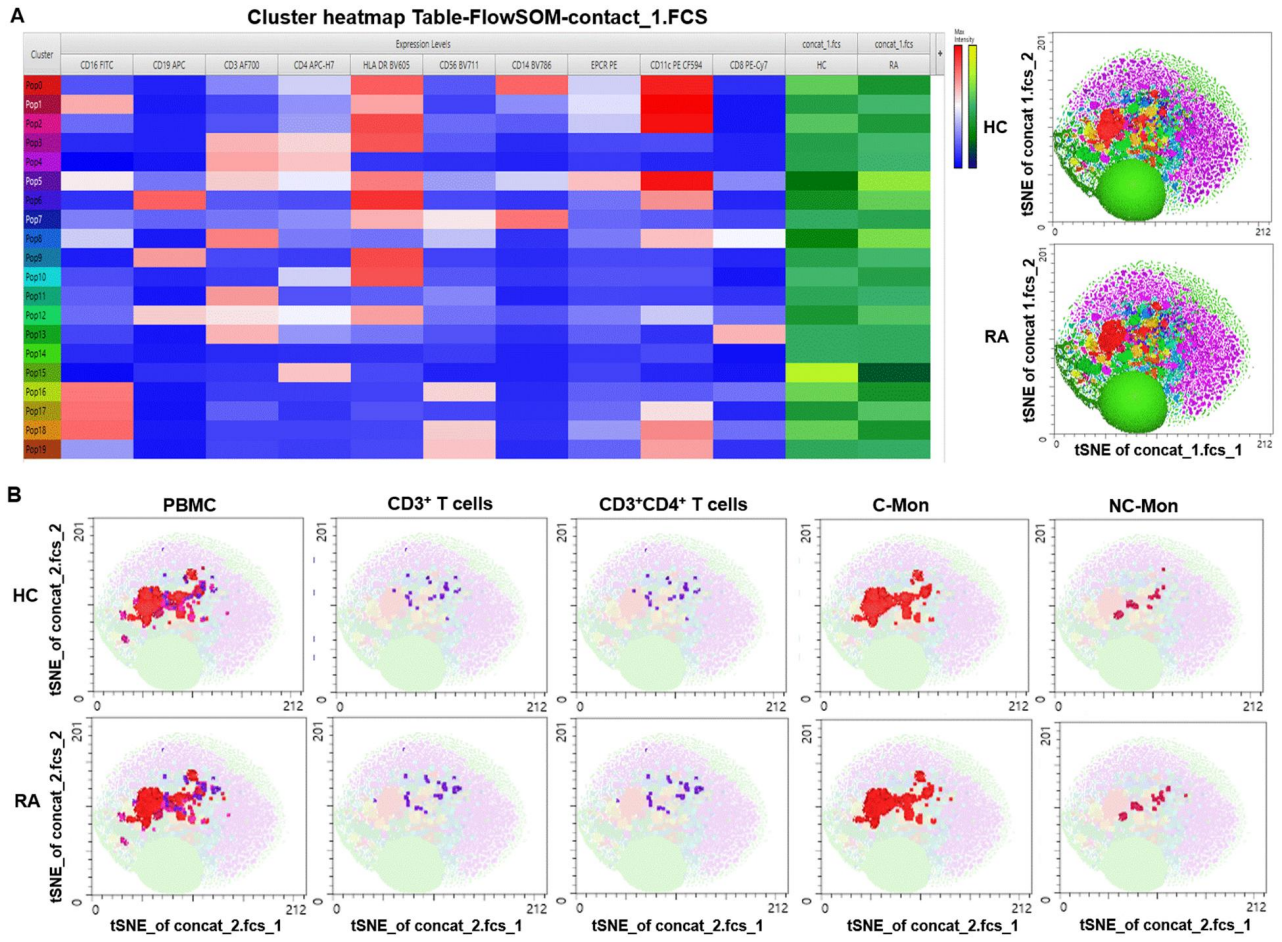
tSNE and cluster analysis of immune cell markers and their mEPCR on paired healthy controls (HC) and patients with RA. (A) The heatmap of FlowSOM and Cluster Explorer analysis of mEPCR and immune cell marker expression on 10 pairs of HC and RA PBMC. (B) mEPCR on immune cells analysed by FlowSOM and cluster in combination with marker enrichment modelling on 10 pairs of HC and RA PBMC. All analysis was performed in FlowJo 10. C-Mon: classical monocytes; NC-Mon: non-classical monocytes

Next, the unsupervised analysis (gated on single cells) using FlowSOM and Cluster Explorer was performed to explore the EPCR expression by multiple immune cells from RA and HC. The heatmap of the EPCR and immune cell marker expression by 10 matched HC and RA was displayed in Fig. 2A. MEM identified that EPCR was expressed mostly by CD3^+^, CD4^+^, CD14^+^, CD16^+^ and/or CD11c^+^ cells, including Pop0 (CD11c + 10CD14 + 6HLA + 5EPCR + 1CD4 + 1), Pop1 (CD11c + 10HLA + 2CD16 + 2EPCR + 1CD4 + 1CD14 + 1), Pop2 (CD11c + 10HLA + 5EPCR + 1CD4 + 1) and particularly Pop5 (CD11c + 10HLA + 3CD3 + 1EPCR + 1CD4+ CD14 + 1CD16 + 1) (Fig. 2A). Using these markers, we identified that EPCR expressing cells were CD3^+^ T cells, including CD3^+^CD4^+^ T cells and monocyte subsets particularly classic monocytes, and confirmed that RA T cells expressed more EPCR than HC cells (Fig. 2B).

### Circulating levels of EPCR are associations with the *EPCR* gene H3 SNP G genotype

**Figure 3.**
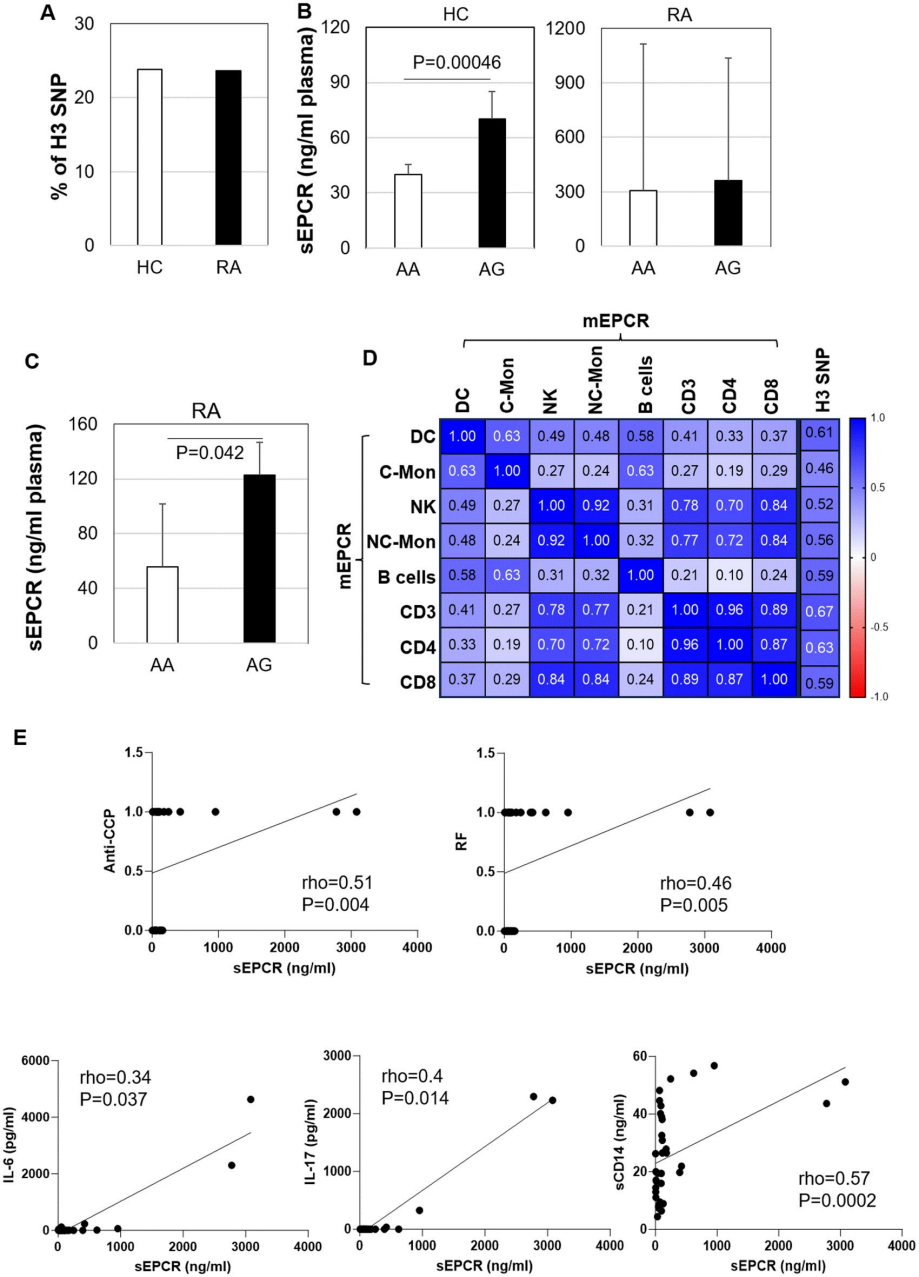
EPCR levels, *EPCR* gene H3 SNP G genotype frequency and their associations in healthy controls (HC) and patients with RA. (A) The frequency of the *EPCR* gene H3 SNP G genotype in RA (*n* = 38) and HC (*n* = 21), detected by PCR and DNA sequencing. (B) sEPCR levels in HC (*n* = 21) or RA patients (*n* = 38) carrying the *EPCR* gene H3 SNP AA and AG genotype (HC, *n* = 5; RA, *n* = 9). (C) sEPCR in age and gender-matched RA patients who carried *EPCR* gene H3 SNP AA or AG genotypes (*n* = 4). Data on the graphs of B&C are shown as mean ± SD. *P* values were obtained by Mann–Whitney *U* test. (D) Correlation matrix of mEPCR on immune cells and *EPCR* gene H3 SNP G genotypes. (E) Correlations of sEPCR with the presence of anti-CCP antibodies and/or RF, sCD14, IL-6 and IL-17 in RA patients. Correlation was detected by non-parametric Spearman’s correlation. Data shown in (D) were Spearman’s rho values, sample sizes were displayed in Tables 1 and 2, rho ≥ 0.35 indicates a *P*-value <0.05. DC: dendritic cells; C-Mon: classical monocytes; NC-Mon: non-classical monocytes; NK: natural killer cells

The levels of plasma sEPCR in RA patients are displayed in Table 1. The *EPCR* gene H3 SNP G genotype has been reported to be associated with increased sEPCR levels [20]. In this study, out of the 21 HC individuals and 38 RA patients analysed, five HC (23.8%) and nine RA patients (23.7%) carried the AG genotype, while no GG genotype was detected (Fig. 3A). The frequency of this SNP G genotype did not differ significantly between RA and HC groups.

**Table 1.** Inflammatory markers/mediators of patients with rheumatoid arthritis

	Anti-CCP	RF	CRP (mg/dl)	ESR (mm/h)	sEPCR (ng/ml)	IL-6 (pg/ml)	IL-17 (pg/ml)	sCD14 (ng/ml)
Overall (*n* = 38)	17+/14−	20+/16−	4.3 ± 6.0 (*n* = 37)	20.5 ± 23.2 (*n* = 36)	301 ± 685 (*n* = 38)	213 ± 862 (*n* = 38)	140 ± 533 (*n* = 38)	27.4 ± 15.2 (*n* = 38)
Females (*n* = 33)	15+/12−	18+/13−	4.6 ± 6.3 (*n* = 33)	22.8 ± 23.8 (*n* = 32)	293 ± 693 (*n* = 33)	221 ± 888 (*n* = 33)	138 ± 548 (*n* = 33)	26.1 ± 14.6 (*n* = 33)
Males (*n* = 5)	2+/2−	2+/3−	3.4 ± 3.8 (*n* = 4)	10.5 ± 10.4 (*n* = 4)	230 ± 405 (*n* = 5)	36.6 ± 54.2 (*n* = 5)	65.4 ± 146 (*n* = 5)	25.2 ± 22.2 (*n* = 5)

The status of anti-CCP antibodies and RF, the levels of CRP and ESR were gathered from The Australian Arthritis and Autoimmune Biobank Collaborative (A3BC), at the time blood was drawn. The levels of plasma sEPCR, IL-6, IL-17 and sCD14 were measured by enzyme-linked absorbance assay. +/−: positive/negative.

In comparison to the *EPCR* gene AA genotype (normal genotypes), HC individuals with the AG genotype had significantly higher levels of sEPCR (*P* < 0.001) as shown in Fig. 3B. Within RA, only under the age- and gender-matched condition, patients carrying the H3 SNP AG genotype had higher sEPCR levels (*n* = 4, *P* = 0.04) when compared with the AA genotype (Fig. 3C).

Table 2 displays the levels of relative RA immune cell mEPCR. Most of them showed positive correlations with each other, with rho > 0.35 indicating significant correlations, as shown in Fig. 3D. Unlike sEPCR, mEPCR levels on all immune cells detected in this study were significantly correlated with the *EPCR* gene H3 SNP, especially on CD3^+^ and CD3^+^CD4^+^ T cells, with rho values of 0.63 and 0.67, respectively (Fig. 3D).

**Table 2.** Levels of cell membrane-bound (m)EPCR on immune cells from patients with rheumatoid arthritis

	DC	C-Mon	NK cells	NC-Mon	B cells	CD3 cells	CD4 cells	CD8 cells
RA (*n* = 34)	3.07 ± 4.04	3.40 ± 3.23	5.79 ± 7.69	3.16 ± 3.48	3.59 ± 3.70	2.09 ± 1.57	1.68 ± 1.26	2.04 ± 2.29
Females (*n* = 30)	3.27 ± 4.26	3.56 ± 3.72	6.02 ± 7.93	3.23 ± 3.64	3.72 ± 3.90	2.22 ± 1.59	1.78 ± 1.25	2.13 ± 2.37
Males (*n* = 4)	1.57 ± 0.99	2.20 ± 1.73	4.10 ± 6.18	1.98 ± 1.96	2.68 ± 1.66	0.96 ± 1.27	1.45 ± 1.71	1.11 ± 1.04

The relative levels of mEPCR were obtained by comparing the flow cytometric data from individual RA samples to the standardized sample in each batch.

DC: dendritic cells; C-Mon:, classical monocytes; NC-Mon: non-classical monocytes; NK cells: natural killer cells.

### Levels of sEPCR in plasma correlate with anti-CCP, RF and inflammatory cytokines in RA

Table 1 shows the plasma levels of sEPCR, IL-6, IL-17, sCD14, CRP and ESR in 38 RA patients. Patients who were anti-CCP and/or RF positive had higher levels of plasma sEPCR (*r* = 0.51, CI: 0.1759–0.7354, *P* = 0.004; rho = 0.46, CI: 0.1485–0.6922, *P* = 0.005; respectively) as shown in Fig. 3E. Levels of sEPCR were also significantly correlated with the levels of IL-6 (rho = 0.34, CI: 0.01349–0.6016, *P* = 0.037), IL-17 (rho = 0.4, CI: 0.07866–0.6417, *P* = 0.014) and sCD14 (rho = 0.57, CI: 0.3037–0.7598, *P* = 0.0002) (Fig. 3E). Levels of sEPCR did correlate with CRP or ESR, or any disease activity measures, nor mEPCR levels (data not shown).

### Levels of mEPCR and the *EPCR* gene H3 SNP G genotype inversely correlate with disease activity scores in RA

**Figure 4.**
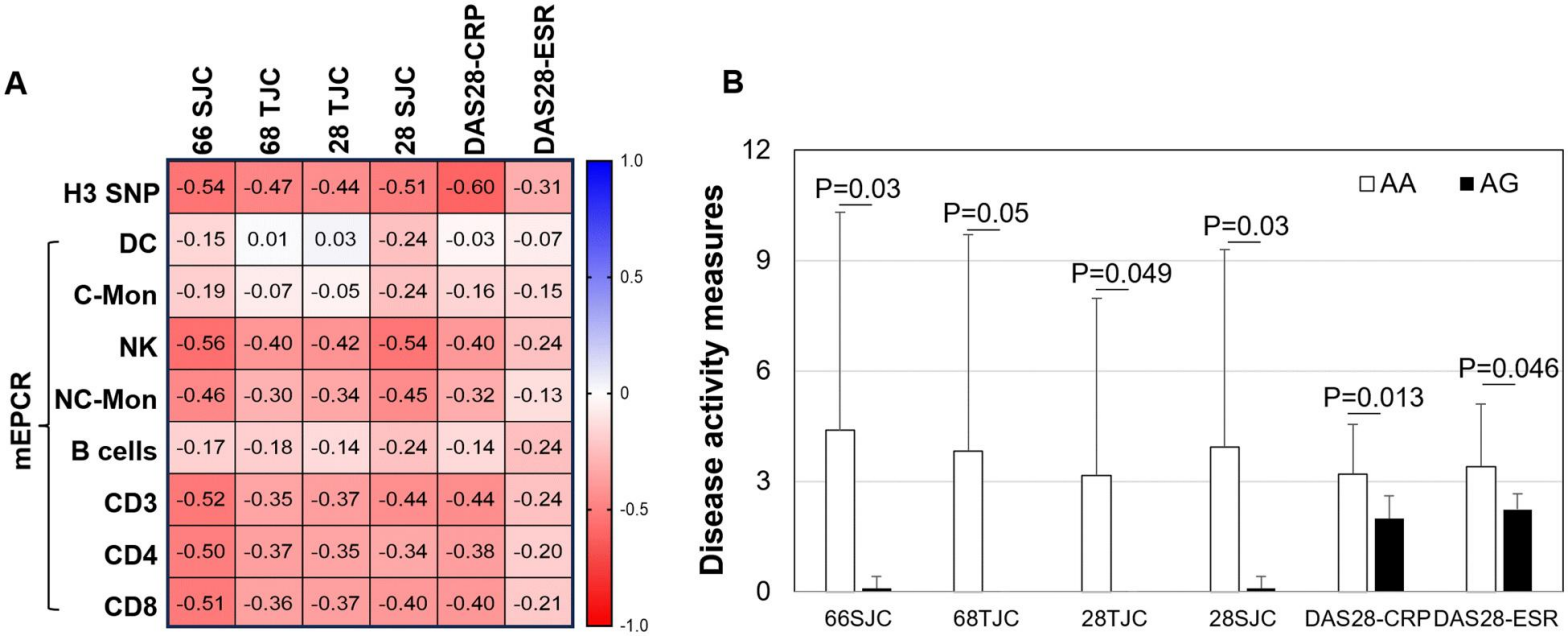
The correlations of mEPCR and *EPCR* gene H3 SNP G genotype with RA disease activity measures. (A) The correlation matrix of immune cell mEPCR, *EPCR* gene H3 AG genotype and disease activity measures in RA patients. Data shown were non-parametric Spearman’s rho values, sample sizes were displayed in Tables 1 and 2, rho ≤ −0.35 indicates a *P*-value <0.05. Correlations were detected by non-parametric Spearman’s correlation. (B) The average disease activity measures of RA patients who carried the *EPCR* gene H3 SNP AA (*n* = 29) or AG (*n* = 9) genotypes. Significance was detected by Mann–Whitney *U* test. Data on the graph are shown as mean (s.d.). SJC: swollen joint count; TJC: tender joint count; DC: dendritic cells; C-Mon: classical monocytes; NC-Mon: non-classical monocytes; NK: natural killer cells

In RA patients, the levels of mEPCR on NK cells, non-classic monocytes, CD3^+^, CD3^+^CD4^+^ and CD3^+^CD8^+^ T cells were inversely correlated to 66/28SJC, 28/68 TJC and/or DAS28-CRP scores (rho < −0.35 indicated significant correlations) (Fig. 4A).

Interestingly, the study also found that the E*PCR* gene H3 SNP G genotype was associated with lower disease activity scores in RA patients. Patients who carried this genetic variant had significantly lower measures for 28/66SJC, 68TJC, DAS28-CRP/ESR when compared patients who did not carry this variant (Fig. 4B). This variant was inversely correlated with RA disease activity measures such as 66SJC (rho = −0.54, CI: −0.7840 to −0.1520, *P* = 0.008), 68TJC (rho = −0.47, CI: −0.7461 to −0.06169, *P* = 0.023), 28TJC (rho = −0.44, CI: −0.7271 to −0.02019, *P* = 0.036), 28SJC (rho = −0.51, CI: −0.7651 to −0.1055, *P* = 0.014) and DAS28-CRP (rho = −0.6, CI: −0.8247 to −0.2166, *P* = 0.004) (Fig. 4A).

Notably, there were no correlations between mEPCR levels and inflammatory markers such as anti-CCP and RF antibodies, CRP, ESR, IL-6, IL-17 or sCD14. These findings indicate that levels of mEPCR and the H3 SNP G genotype of the *EPCR* gene may serve as specific markers for measuring RA disease activity.

## Discussion

This study aimed to investigate the relationship between EPCR and inflammatory markers/cytokines and disease activity in patients with established RA. Our preliminary findings indicate that the levels of circulating sEPCR were positively associated with inflammatory markers/cytokines, whereas mEPCR on T cells and NK cells was inversely correlated with disease activity measures in RA. Furthermore, in patients with RA, the *EPCR* gene H3 SNP G genotype was linked to higher levels of mEPCR on immune cells and lower disease activity measures. It appears that mEPCR levels on immune cells, particularly on T cells and NK cells, and *EPCR* gene H3 SNP G genotype may have protective roles. In contrast, once cleaved, it may contribute to inflammation in RA.

This study has found that RA patients had higher levels of plasma sEPCR compared with HC and it had positive correlations with the presence of anti-CCP antibodies and RF, and the levels of plasma inflammatory cytokine sCD14, IL-6 and IL-17. In RA, the presence of RF and anti-CCP antibodies predicts a more aggressive and destructive course of the disease [36], whereas IL-6 and IL-17 have positive correlations with RA disease activity [37]. Plasma sEPCR levels were also linked to sCD14, which has been shown to reflect RA disease activity and can predict the treatment response of methotrexate and bDMARD, with responder RA patients exhibiting lower sCD14 levels [38]. Furthermore, sCD14 can induce inflammation and proliferation of RA synovial fibroblasts [39], the key driver of joint inflammation and destruction. These results indicate the inflammatory effect of sEPCR in RA.

This study has demonstrated that higher levels of mEPCR on NK cells and T cells, particularly CD3+CD8+ T cells, were associated with lower disease activity measures in established RA (Fig. 4). This finding is consistent with previous studies which have also indicated that T cell-specific deficiency of EPCR resulted in the exacerbation of experimental autoimmune encephalomyelitis in mice [17]. Furthermore, EPCR-deficient mice are more susceptible to dextran sulphate sodium-induced colitis, which is characterized by inflammation and mucosal barrier disruption [40]. However, it is worth noting that EPCR deficiency protects against collagen-induced arthritis [34] and bleeding-induced joint injury [28]. Paradoxical findings like these also appear in cerebral malaria (CM), where EPCR overexpression is associated with severe CM [25], but endothelial EPCR expression is significantly decreased in cerebral blood vessels from patients with CM [41]. The underlying mechanisms of these conflicting functions of EPCR are not clear, but they may be associated with its cell or ligand-specific function [3]. For instance, mEPCR on murine CD3+CD4+ T cells suppresses the generation of pathogenic Th17 cells [9], while EPCR expressed by RA synovial fibroblasts promotes the invasion of these cells [42]. The binding of APC to EPCR has anti-inflammatory, antiapoptotic, and barrier-protective properties. On the other hand, the binding of sPLA2V to EPCR promotes endothelial cell apoptosis [15], as well as the proliferation and invasion of RA synovial fibroblasts [42]. Additionally, a recent study found that EPCR can bind with aPLs, leading to prothrombotic and inflammatory responses and promoting mouse foetal loss and thrombosis [14]. RA patients have significantly higher levels of aPLs [43–45] and sPLA_2_V [46, 47] when compared with the general population. Therefore, the functions of EPCR in specific diseases may depend on the balance of multiple ligands and the specificity of tissues/cells.

The H3 SNP genotype of the human *EPCR* gene, including AG and particularly GG genotype, has been linked to increased levels of plasma sEPCR [20], which explains around 85% of the phenotypic variance [20]. This association was observed in HC individual, but not in those with RA (Fig. 2). Inflammatory mediators such as TNF and IL-1 can induce EPCR shedding [3]. Therefore, the higher levels of sEPCR in individuals with RA are likely partly resulting from these mediators as well.

RA patients who carried with the *EPCR* gene H3 SNP G genotype appeared to have lower disease activity measures. This SNP also demonstrates a protective effect in severe malaria in adults [48]. The reason for this may be that the *EPCR* gene H3 SNP G genotype could lead to increased plasma levels of PC [49]. This, in turn, allows more PC to bind to EPCR on the cell surface in these RA patients compared with those with AA genotypes. As a result, the levels of APC are increased, which can protect against severe RA [33]. Furthermore, higher levels of sEPCR induced by H3 SNP G genotype may bind to other inflammatory mediators like sPLA2V [15, 42] and reduce their damaging effect on RA.

The present study has some limitations. Firstly, our sample size was relatively small. A larger patient cohort would have provided a more precise estimation of the interaction effects, with narrower confidence intervals. Secondly, we did not examine the potential influence of RA treatments on EPCR expression/shedding due to the small sample size and the many different types of drugs patients used in our study. Inflammatory mediators in RA, such as TNF-α, IL-1β and IL-6, can affect EPCR expression and shedding [21, 22]. Treatments targeting these inflammatory mediators may significantly impact the levels of EPCR in the circulation of RA patients. For instance, anti-TNF-α therapy has been shown to reduce EPCR expression on T cells in psoriasis patients [29]. Further research on the effects of these medications on EPCR would help to better understand the role of EPCR in this disease. Finally, patient selection was based on the availability of a single centre, which could potentially create a selection bias due to the small number of patients analysed and the fact that they were all treated in a single centre. As a result, our observations should be considered preliminary and should be validated in a larger and more diverse independent patient population.

In conclusion, this pilot study suggests that sEPCR may have an inflammatory effect, whereas the *EPCR* gene H3 SNP G genotype and immune cell mEPCR may play protective roles in RA. Further prospective studies are necessary to confirm and validate these findings, and to investigate the role of EPCR in the disease and treatment course of RA.

## Supplementary Material

rkae096_Supplementary_Data

## Data Availability

The data supporting this article will be shared on reasonable request to the corresponding author.
