# Supplementary material for: Exploring the association between circulating endothelial protein C receptor and disease activity of rheumatoid arthritis in a pilot study
Source: Rheumatol Adv Pract. 2024 Aug 6;8(3):rkae096. doi: 10.1093/rap/rkae096 (PMC11343369; doi:10.1093/rap/rkae096)
Supplement: rkae096_Supplementary_Data [file rkae096_supplementary_data.docx]

**Supplementary Table S1 The characteristics of patients with rheumatoid arthritis.**

| **Sex** | **Age** | **Disease duration**  **(years)** | **Anti-CCP** | **RF** | ***CRP**  **(mg/L)** | ***ESR**  **(mm/hr)** | **csDMARD status** | **b/tsDMARD status** | **Glucocorticoids (current)** |
| --- | --- | --- | --- | --- | --- | --- | --- | --- | --- |
| F | 38 | 24 | + | + | 1.5 | 15 | Current | No | Yes |
| F | 62 | 0 | + | + | 12 | 41 | Current | No | Yes |
| M | 65 | 0 | − | − | 2.3 | 7 | Current | No | No |
| F | 62 | 17 | UNK | + | 0.4 | 1 | Current | Yes | No |
| M | 37 | 2 | − | − | 9 | 26 | Current | Yes | Yes |
| F | 63 | 44 | − | − | 8.7 | 51 | UNK | Yes | Yes |
| F | 66 | 30 | + | + | 3.1 | 28 | Current | Yes | Yes |
| F | 79 | 15 | + | + | 0.8 | 6 | Past | Yes | No |
| F | 52 | 19 | + | + | 11 | 120 | Current | Yes | Yes |
| F | 21 | 19 | − | + | 3.8 | 7 | Current | Yes | No |
| F | 72 | 30 | + | + | 30 | 40 | Current | Yes | No |
| F | 49 | 21 | − | − | 4.5 | 9 | Past | Yes | No |
| F | 70 | 8 | − | + | 2.7 | 30 | UNK | No | No |
| F | 69 | 1 | − | − | 17 | 33 | Current | No | Yes |
| F | 59 | 13 | UNK | − | 0.7 | 25 | Past | Yes | UNK |
| F | 40 | 8 | + | + | 0.4 | 7 | UNK | Yes | No |
| F | 73 | 18 | + | + | 0.4 | 20 | Past | Yes | No |
| F | 65 | 37 | − | − | 0.7 | 26 | Past | Yes | No |
| F | 66 | 19 | + | + | 1.3 | 19 | UNK | No | No |
| F | 40 | 28 | − | − | 2.1 | 5 | Current | Yes | No |
| F | 80 | 30 | UNK | UNK | 0.9 | 6 | UNK | Yes | Yes |
| F | 35 | 10 | + | − | 0.7 | 46 | UNK | Yes | UNK |
| F | 42 | 11 | − | − | 1.6 | 6 | Current | Yes | UNK |
| F | 66 | 1 | + | + | 4.3 | 9 | Current | No | No |
| F | 31 | 5 | − | − | 1.4 | 11 | Past | Yes | No |
| F | 33 | 0 | + | + | 0.3 | 4 | UNK | No | No |
| F | 31 | 15 | + | + | 0.5 | 2 | Past | Yes | UNK |
| F | 42 | 3 | − | − | 0.7 | 0 | Past | UNK | No |
| F | 65 | 0 | − | − | 5.7 | 7 | Current | No | Yes |
| M | 68 | 4 | + | + | 1 | 6 | Current | No | Yes |
| F | 72 | 13 | + | + | 4.3 | 3 | Current | Yes | No |
| F | 79 | 0 | + | + | 8 | 46 | UNK | No | Yes |
| M | 39 | 0 | + | + | 1.2 | 3 | UNK | No | No |
| M | 29 | 0 | + | − | 3.0 | 6.0 | UNK | No | No |
| F | 60 | 15 | + | + | 4.8 | 13 | Current | Yes | UNK |
| F | 60 | 17 | + | + | 2.2 | 52 | Current | No | No |
| F | 38 | 3 | + | − | 1.3 | 8 | Past | Past | No |
| F | 36 | 14 | − | − | 15.3 | 32 | Current | Yes | No |

*Most recent data, which was gathered from The Australian Arthritis and Autoimmune Biobank Collaborative (A3BC), at the time blood was drawn. These values were not always measured on the same day as the blood sample collection for the A3BC biobank. Anti-CCP: anti-cyclic citrinullated peptide; b/tsDMARD: biologic or targeted synthetic disease-modifying antirheumatic drugs; CRP: C-reactive protein; csDMARD: conventional synthetic DMARD; ESR: erythrocyte sedimentation rate; F: female; M: male; UNK: unknown; RF: rheumatoid factor; +: positive; −: negative.

**Supplementary Table S2 The demographic and clinical parameters of patients with rheumatoid arthritis.**

|  | **Age**  **(years)** | **Duration (years)** | **66 SJC** | **68 TJC** | **28 TJC** | **28 SJC** | **DAS28-CRP** | **DAS28-ESR** |
| --- | --- | --- | --- | --- | --- | --- | --- | --- |
| Overall  (n=38) | 54.9±16.9 (n=38) | 13.1±12.0 (n=35) | 4.0±5.7 (n=37) | 3.6±5.7 (n=37) | 2.8±4.5 (n=37) | 3.4±5.2 (n=37) | 3. 0±1.4 (n=32) | 3.3±1.6 (n=32) |
| Females  (n=33) | 55.0±16.6 (n=33) | 14.8±11.4 (n=33) | 3.9±5.6 (n=32) | 4.6±7.5 (n=32) | 3.5±5.4 (n=32) | 3.5±5.1 (n=32) | 2.9±1.5 (n=29) | 3.3±1.6 (n=29) |
| Males  (n=5) | 47.6±17.7  (n=5) | 1.2±1.8  (n=5) | 5.2±5.9  (n=5) | 4.4±6.2  (n=5) | 2.4±2.3  (n=5) | 3.2±1.7  (n=5) | 3.2±1.3  (n=3) | 3.2±1.7  (n=3) |

CRP: C-reactive protein; ESR: erythrocyte sedimentation rate; SJC: swollen joint count; TJC: tender joint count**.**
